# Supplementary material for: Patterns of Intron Gain and Loss in Fungi
Source: PLoS Biol. 2004 Nov 30;2(12):e422. doi: 10.1371/journal.pbio.0020422 (PMC532390; doi:10.1371/journal.pbio.0020422)
Supplement: Table S1 — Also available at http://genes.mit.edu/NielsenEtAl/. (4.3 MB ZIP). [file pbio.0020422.st001.zip › NielsenEtAl/html/1432.html]

AN1377.1.NCU03937.1.MG00990.1.FG09283.1


```
 CLUSTAL W (1.82) Multiple Sequence Alignments - Introns Inserted


Sequence 1: NCU03937.1	1232 aa
Sequence 2: MG00990.1	1257 aa
Sequence 3: FG09283.1	1496 aa
Sequence 4: AN1377.1	1255 aa
Alignment Length: 1604 aa
Number Identitical Residues: 505 aa
Alignment Score (without introns) 25932


MG00990.1 	-------------------------------------------------~----------
NCU03937.1	-------------------------------------------------~----------
FG09283.1 	MTEHLPNKQKGRERDRIKRTKDGVTQLPRKKFYRQRAHANPFSDHMLEY2PKSPDHMDWS
AN1377.1  	-------------------------------------------------~----------
          	                                                            

MG00990.1 	----------------------------------------------------~-------
NCU03937.1	----------------------------------------------------~-------
FG09283.1 	PYFPHYVQEGTSEDSSKPPRLTKDVEIVDIGCGFGGLTVALAPKMPDTLTLG1LEIRTQV
AN1377.1  	----------------------------------------------------~-------
          	                                                            

MG00990.1 	------------------------------------------------------------
NCU03937.1	------------------------------------------------------------
FG09283.1 	AGYVQERIKALRSQNSDNMYQNVACIRANTMKFLPNFFRKAQLSKIFICFPDPHFKARKH
AN1377.1  	------------------------------------------------------------
          	                                                            

MG00990.1 	------------------------------------------------------------
NCU03937.1	------------------------------------------------------------
FG09283.1 	KARIVSTTLNSEYAYALRPGGIVYTITDVEDLHLWMVQHLEAHPAFERISKEEEEADECV
AN1377.1  	------------------------------------------------------------
          	                                                            

MG00990.1 	------------~------------------------------------MAPLETKELAL
NCU03937.1	------------~-------------------------------------MVLQGRDIAL
FG09283.1 	HVMSTETEESKK0LVQSGLDLHYLLSHHRLYHLHPSIFVQECGFEVNIPVMALKRRDVIV
AN1377.1  	------------~-------------------------------------MPLKHRDYAL
          	                                                    *: ::  :

MG00990.1 	AALGSFVAWGYATHWFPPLRLAGYAFVAGAVLAVLSLAALVLLTSRGAQYRTHTTSSRRP
NCU03937.1	AAIAAFISWGYAVHWFPAIRWANYAFAAGVLVTLVGLLALLMLTSRGSHHHRLRPPPR-P
FG09283.1 	AGLAAFIAWGFAASWSPVLRWAGHAFVAGSLVTLVLLLTGLFLVSRRPNDRTLP-----P
AN1377.1  	AGLGGFIAWGFAIHWLPVLRYLGYALALGAFLASSAIFGLVLLTTRNLPSSKFTRP--KT
          	*.:..*::**:*  * * :*  .:*:. * .::   :   ::*.:*         .   .

MG00990.1 	HGVAFVAPAAWPAELAALESRQSYDPDPIAPDTPKVSEAVEEVLGYIVRDFVQSWYGSIS
NCU03937.1	HGAAFVAPGSWSKEVAALRARQTSTKAPLYPESPKVSAALDEVLGYVIRDFIQVWYSSIS
FG09283.1 	NGVTFLNPKSWRSEVQALRQRQSYTPTQIQPESPRVSKAIDDLLGLIVRDFVSSWYSNIS
AN1377.1  	LPVAFVSRRHWKNEVQRYGKRAEYHAESLYPESFIVSESLNEILSFITRDFIASWHRSIS
          	  .:*:    *  *:     *       : *::  ** :::::*. : ***:  *: .**

MG00990.1 	KNPVFTNEVDRAIRAALGNIRDRILQVDLAEVLTSRLVPILTAHFRGFYDAERAVRGKKL
NCU03937.1	KNPVFTDEVDKAIRGAILTVRDRLAQLDLATILTTRLVPILTAHFRDVYEAEKSVRGRKL
FG09283.1 	RNPTFTNQVDKAVREALLSLCDILRDKDLADLVTSRLVPLLTAHFRDFYEAEKSIRGKKL
AN1377.1  	SNPAFPNEVDKVIRTAVGNLRSRLLAEDIVSLVVSRIFPILTTHLKEFDIAERSVRGRNL
          	 **.*.::**:.:* *: .: . :   *:. ::.:*:.*:**:*:: .  **:::**::*

MG00990.1 	NRSVTETDELDLAIAAKYNDGKLHPAAALSHMDAKTTQQEYLRDVVAKLLPKVLPENMSR
NCU03937.1	NRSVTETDELDLAIASKYRDGKLHPAATLSFSDTKTTQQDYLRKVMVRILPKILPKSMLA
FG09283.1 	NRSVTESEELDLAIASKFRDGRLHPAASLSFPDTKMVQQDYLRSLVAKIMPQILPGNMLS
AN1377.1  	TRNVTESEELDLAIAKKYRDGQLHPAAVLSLSDQKAVEQEYLRKVTVGLLPQLFPANVLN
          	.*.***::******* *:.**:***** **  * * .:*:***.: . ::*:::* .:  

MG00990.1 	SRAVTTLVREIVACAVLGSVMQILSDPDTWNQVMENYGRSMLQDRSTVRKLRAALDQHAS
NCU03937.1	SGAVSAIVREITACAVMFPIMRMLSDPDTWNQLMENYGRSMLQDRSTVRKLRAALDQHAS
FG09283.1 	SRAVSIIIREIVGCSVLFPVIQLLSEPDTWNQLMENMGRSMLQDRSTVRKLRAALDQHAP
AN1377.1  	SRIVSVLIREILSCAVLFPLVSVASDPDTWNQLIEAYGRTTLQDRKTVRKLRAALDEHAS
          	*  *: ::*** .*:*: .:: : *:******::*  **: ****.**********:**.

MG00990.1 	PAPRTSKPVAFPRLSPGDSERRFEKFVRSVRKVSNLSDARRFRSEVASQLKRESQKEGQD
NCU03937.1	PAPRVNKPVAFPRMIPGDNERKFEKFIRAIRKTNNLSDARRFRSEVAIQIKRDSQQENVD
FG09283.1 	STPRSNKVASMPRVAPGDSERKFEKFIRAIRKVNNLSDARRFRSEVASQLKRDSLQDNQD
AN1377.1  	PAPKAKRGHPFPRLSPNDSERAFERFVRTIRRCNNLSDARRFRALIASQLKRESMVEGQD
          	.:*: .:  .:**: *.*.** **:*:*::*: .*********: :* *:**:*  :. *

MG00990.1 	PVHLRRLEMGKRLLDQRVQQLAAAGGDRRAAQVPMPSQAPPRASRLENASLEELLRDSSG
NCU03937.1	QVYLRRLEMGKRLLDQKVHHLAAGG-DRRALPPQTTALPTPTVSRLENASLTDVLRDPSS
FG09283.1 	QVYLRRLEMGKRLLDQRVTHLAAGG--ERRPPGPLPLPSTSSKSKLESAPLVDFLRDPSA
AN1377.1  	PVYLRRLETGKRVLDQKVAKLSAPNDQTSHVAAAASHFRRKSSSATQEASLVDVMHDASG
          	 *:***** ***:***:* :*:* ...        .       *  :.*.* :.::*.*.

MG00990.1 	LSYFMEYMDRQRLLPLVQFWLVVDGFRNPLEDDGPEDEQLPLVLPPWNNSDRLDLAQINQ
NCU03937.1	LSYFMEYMDRQRLMPLVQFWLVVDGFRNPLEDDGPEGEQLPLQLPPWTDSDRLDLAQIEA
FG09283.1 	LSYFMEYMDRQNLMPLVQFWLVVDGFRNPLEEDG-HDDELPSTLPMWTDSDRQDLQQINQ
AN1377.1  	LSYFMEFMDRQHLMSLVQFWIVVDGFRNPLEDDF--GDETSPSSNTWTTADRNDIALISA
          	******:****.*:.*****:**********:*   .:: .     *. :** *:  *. 

MG00990.1 	AYLSMPVLKVPPSSRKLVQEFLDAGSKAIPAQYYRARRAILRAQTAVLDEMRINHFQNFK
NCU03937.1	AYLSRPELKVPDSSKRTIQEFLRAGRRASPEQYYKARRAVLRAQSAVLEEMKAKHFGNFR
FG09283.1 	AYLSRSELKVPDHSKNEVKEFIKAGKTATPMQYYRARQAILKAQSAVLEEMRARFFQGFK
AN1377.1  	TYLAKPELKVTEESRQVVKAFLSAGKRATPEQYRKARTVILSTQSAILEELQEVYFPKFK
          	:**: . ***.  *:. :: *: **  * * ** :** .:* :*:*:*:*::  .*  *:

MG00990.1 	KSDLFYKCLAAEEAASRIAAPYTPAPAGQTLTAAPTPTSPALNRASQSFTTARPNHATRL
NCU03937.1	KSDLFYKALASEEASKSMVAPVS----------GSSTPGPSVARASS--FTSKPNPQLRM
FG09283.1 	KSDLFYKCLAAEEAANNQTLRSTP---------QPEAQTLAPGRTSKT-LPAKPRPVSRL
AN1377.1  	QSDLYWKYLASDDVSHGSAAMSS-----------PSSQQSLVGSLDAQERRPLPLLLNRT
          	:***::* **:::.:   .   :           . .        .     . *    * 

MG00990.1 	ANRLNP-VPNRRTGSTSDLRSLTVNGSGLDPLTSSRRSLDEGNSSPLFGDEEDWDNEALA
NCU03937.1	ALRLPPGARNRGAGSSLDIRAGTQS---IDALMGTRRSMEDDSSITPMFDDDDLGEDGLA
FG09283.1 	TTQLSN--TSKRTGSASDLRSLNFNG--NGHGHRSRRSLDEG-SLTPLFDDDDIDTDGLG
AN1377.1  	ASQPGPKAKVDLRRAAISSNDVSGLGKLFDGEESTRRSMDSERSAPLFDDDYDTDPLALS
          	: :           :: . .  .  ..  .    :***::.  * . : *: * .  .*.

MG00990.1 	DSVQSLDQEPMTAPPPDTRVVKAVEDALSTIMEDSQPPTAEDLRASLFSGEDSSSGIFSP
NCU03937.1	DSVQSLEQEP-APPVPDTRVVQAMEQALNNIMEDSQPPTAEDLRASLFGDDGNNSSLFSS
FG09283.1 	DSVQSLDAESNSQQLPDTQVVQAVEEALTNILENDRPQTAEDLRASLFENGGSSSIGGLD
AN1377.1  	SQSLGQDTPNGGPEISERRVIENMEAALNDIITN----VPKNGRIEELSTSDIHQGSPRN
          	..  . :        .: :*:: :* **. *: :    ..:: * . :   .  .     

MG00990.1 	ETES--LRG------SARGSLDISRPGQ---------PAKEVEKPSLSSLGLVSAASRIG
NCU03937.1	PLKSGPLRAMSATPRSNHGSVDLGRPSRGSADMPRPMTAKEPEKPSLASLGLVSAASRIG
FG09283.1 	DNASSLFSG--HDNESHRGSLDMGLRPG--------ALVKEGEKPSLSSLGLVSAASRIG
AN1377.1  	SNESSRVEG--------RG--------------------DDKLKPSIASLGLVDGSSRRG
          	   *. . .        :*                    .:  ***::*****..:** *

MG00990.1 	VFEDNDLFADEDKHLSDEREDLDGGGEDDDIDEVHEAAPGDLGLAEAITALSNDIDKLVA
NCU03937.1	VFIDDDLFADENRFLSDEHEYSDENKDDDEIDEVHEAAPGDLGLAEAITVLSNDIDRLVA
FG09283.1 	VFVDDDLFGDENKDLPDDGD-PDEGPPPDDEDEVHEAAPGDLGLAEAITALSNDIDRLVA
AN1377.1  	VF-DDDLFPEQQNYLEDEYEEPEGTAGKYSALEVHEAAPGDLGLTEAIQALTADIEKLGS
          	** *:*** :::. * *: :  :      .  ************:*** .*: **::* :

MG00990.1 	QEAIVESLQRKAELTNNTAELRILRKSKASLQREIRRKELQRQQYVIQESDNSLYGRSTI
NCU03937.1	QDAVVDSLLKKAELTNNTAELRILRKSKASLQREIRRKELQRQQYVIQESDNSLYGRSTI
FG09283.1 	QEAVVESLTRKAELTNNTAELRILRKSKASLQREIRRKELQRQQYVIQESDNSLYGRSTI
AN1377.1  	QEAVVDTLTRKAELTNNTAELRILRKSKMSIQRELHRKEMQRQQYMIQESDNSLYGRSTV
          	*:*:*::* :****************** *:***::***:*****:*************:

MG00990.1 	KITGIQVGRDDDGKEYALY~AIEVSRDAGEKMPAATWVVTRRYSEFLELHQKLRSRYPSV
NCU03937.1	KIKNIHVGREEDGKEFAMY~AIEVLRNAGEKMPAATWTVTRRYSEFLELHQKLRSQYSSV
FG09283.1 	KIKSIQVGREEDGREFALY~VIEVQRNAGEQMPAASWVISRRYSEFHELHQKLRSRYPSV
AN1377.1  	RIKSIVVGKEEDGREFAMY1VIEVQRNAGEQMPAGSWVVARRYSEFHELHHQLRTRYPSV
          	:*..* **:::**:*:*:* .*** *:***:***.:*.::****** ***::**::*.**

MG00990.1 	RNLDFPRRRMVMKLQSEFLQKRRDALEKYMRELLLLPEACRSRDLRAFLSQTGVSGAGAA
NCU03937.1	RNLDFPRRRMVMKLQNDFLQKRRVALEKYLNQLLLLPDVCHSRELRTFLSQSTITPTTAT
FG09283.1 	RNLDFPRRRMVMKFQSEFLRKRRTALEQYLQDLLLLPEACRSRELRAFLSQSVIT----Q
AN1377.1  	RQLEFPRRRMVMKLQKEFLQKRRLALEAYLQHLLLLPEVCRSRDLRAFLSQRAIIP----
          	*:*:*********:*.:**:*** *** *:..*****:.*:**:**:****  :      

MG00990.1 	GRDLVDRED-KKDMMTRLYDSVADGMEDILGSIPVLDQLSLAGQNLIAAATSQLNNTVPL
NCU03937.1	HGQTSSLEDSKKDMMTRLYDSVADGMEDILGNIPVLDQLSLAGQNLLAAATNQQLAAASA
FG09283.1 	GQDILDRED-KKDMMTRLYDSVADGMDDILGNIPVLDQISEAGQSLIAAATNQLN-TVPL
AN1377.1  	-RDETPRDGETKDLVTRIYNSVADGMDDFLGNFGVLDQLSIAGQNLISAATNQQQPNTSA
          	  :    :...**::**:*:******:*:**.: ****:* ***.*::***.*    .. 

MG00990.1 	GGGSGGVVGADEGGMSAAEAEAELNAFE---NKELEPFVKPICDVFLEVFELNRGNNWLR
NCU03937.1	AGAGG------DDVVTAAEAEAELNAFELTNDKELEPFVKPICDIFLEVFELNRGNNWLR
FG09283.1 	N--------VNEENFPAAEAEAELNAFE---NKELEPFIKPICDIFLEIFELNKGNNWLR
AN1377.1  	VSTDSG--LTTEDAVTTAEAEAELNAFE---DRELEPFIKPICDLFLETFELNKGNNWLR
          	 . ...     :  ..:***********   ::*****:*****:*** ****:******

MG00990.1 	GRAVVVVLHQLLGGTIERKLRDNVRLLVQQDAVLKYVGLVRESMWPGGCSATQRPAKAPR
NCU03937.1	GRAVVVVLHQLLGGTIERKLRDNVRMLVAEDALLRYINLVKDGMWPSGELASN---KPPR
FG09283.1 	GRAVVVVLQQLLGGTIERKVRDNVKMLAQDENVLKCISLVQDSLWPGGELQRD---RKPR
AN1377.1  	GRAVVVVLHQLLGGTIERKVREGARSLFQDEHLLRYLTLAKDTLWPGGVLREN----RVR
          	********:**********:*:..: *  :: :*: : *.:: :**.*    :      *

MG00990.1 	TPAQKSRTRTEASLMLATLVPDLAGSVVGRLNAQAASRRIFATLNNQRLN2THLVYTFLD
NCU03937.1	TAQEKARTKTEASLMLATLVPDLAASVVGRLNAQAASRRIFATFNNERLK~---------
FG09283.1 	TAAEKKKTRTEASLMLATLVPDLAGSVVGRVNAQAASRRIFATLNNSRLK~YDIR-----
AN1377.1  	TASERLKSRTEASLVLATLVPELAGNVVGRANAQAAARRIFATLNNQRLN~AHLIFTLLD
          	*. :: :::*****:******:**..**** *****:******:**.**:      :  .

MG00990.1 	EIIDILFGEE--------~-------~------------------------
NCU03937.1	------------------~-------~------------------------
FG09283.1 	------------------~-------~------------------------
AN1377.1  	EIVLVLFGGEKVTGRTRL2LQDDKVF0SSFDGLDGLDTLSSGGMSSVTNND
          	.      . .. :. :    ....   :: .. .. .: ::.. :: :...
```
